# Fas Versatile Signaling and Beyond: Pivotal Role of Tyrosine Phosphorylation in Context-Dependent Signaling and Diseases

**DOI:** 10.3389/fimmu.2016.00429

**Published:** 2016-10-17

**Authors:** Krittalak Chakrabandhu, Anne-Odile Hueber

**Affiliations:** ^1^Univ. Nice Côte d’Azur, CNRS, Inserm, iBV, Nice, France

**Keywords:** apoptosis, Fas/CD95, survival signals, tyrosine phosphorylation, disease

## Abstract

The Fas/FasL system is known, first and foremost, as a potent apoptosis activator. While its proapoptotic features have been studied extensively, evidence that the Fas/FasL system can elicit non-death signals has also accumulated. These non-death signals can promote survival, proliferation, migration, and invasion of cells. The key molecular mechanism that determines the shift from cell death to non-death signals had remained unclear until the recent identification of the tyrosine phosphorylation in the death domain of Fas as the reversible signaling switch. In this review, we present the connection between the recent findings regarding the control of Fas multi-signals and the context-dependent signaling choices. This information can help explain variable roles of Fas signaling pathway in different pathologies.

## Introduction

Fas (TNFRSF6/CD95) belongs to the tumor necrosis factor receptor superfamily. When bound to Fas ligand (FasL) or agonistic antibodies, Fas can recruit Fas-associated death domain-containing protein (FADD), procaspase-8, procaspase-10, and cellular FLICE inhibitory proteins (c-FLIPs). This leads to the formation of the death-inducing signaling complex (DISC), the caspase cascade and ultimately apoptosis (1, 2). Apoptosis mediated by the Fas/FasL system is essential for shutting down chronic immune responses (3–5) and preventing autoimmunity and cancer (6). The downregulation of Fas in some cancers prompted the opinion that it was a tumor suppressor. However, while Fas is often downregulated in cancer, it rarely is completely lost (7). Moreover, Fas also mediates cell survival, proliferation, and motility, which can promote autoimmunity, cancer growth, and metastasis (7–12).

Current Fas-targeting therapies aim to activate or inhibit Fas signaling (13, 14). However, without understanding when and why Fas assumes different roles in different pathological contexts, these therapies face a major challenge.

Physiologically, the presence of different FasL forms is an important extrinsic factor that can influence Fas signaling modes. Membrane-bound FasL is essential for activating Fas-mediated apoptosis and thus instrumental in the safeguard against autoimmunity and cancer. Meanwhile, excess soluble FasL (sFasL) may promote autoimmunity and tumorigenesis through non-apoptotic activities (6). However, knowing the different functions of FasL does not sufficiently describe how Fas ultimately takes the apoptotic or non-apoptotic role.

Fas possesses the protein-interacting domain, death domain (DD) (15, 16). Fas multi-signaling requires an efficient molecular switch in DD allowing different signaling complex formations. This agrees with the observation that most disease-causing mutations are in DD (17, 18). This review discusses the regulation of Fas multi-signaling at DD level by tyrosine phosphorylation and implications in human pathologies.

## Death Domain

**Figure 1:**
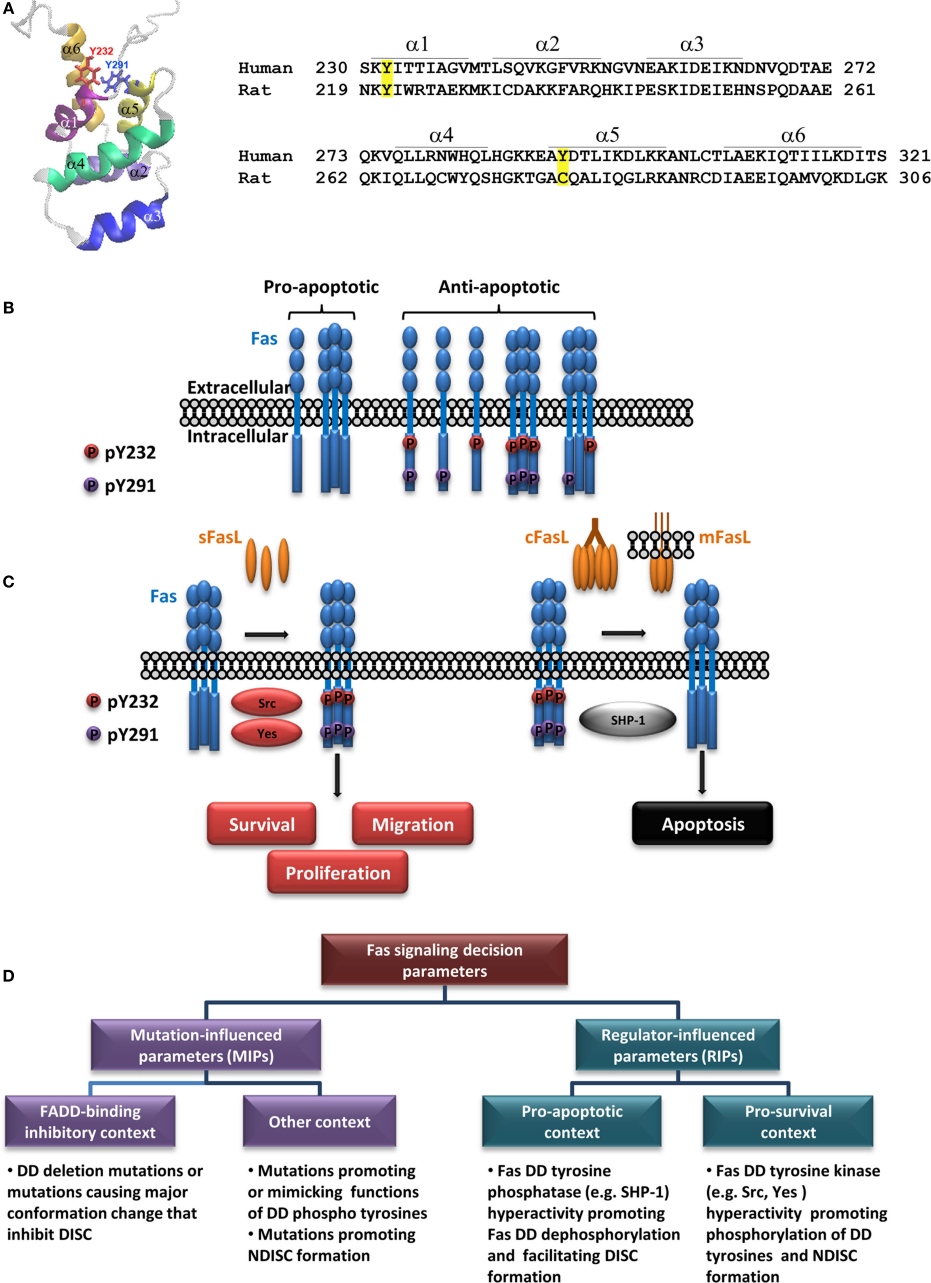
**Regulation of Fas multi-signaling by death domain tyrosine phosphorylation and pathologies**. **(A)** Fas death domain structural model demonstrating six antiparallel α-helices (PDB: 1DDF) (19) with the side chains of Y232 and Y291 indicated (left) and amino acid sequences of human Fas and rat Fas encompassing the death domain with positions of each helix indicated (right; α, α-helix). Tyrosine phosphorylation sites in hFas and corresponding residues in rat Fas are highlighted. Amino acid numbering is according to UniProt entries P25445 and Q63199. **(B)** A diagram depicting different states of Fas, with respect to its ability to transmit apoptotic signal, as affected by its death domain phosphorylation [adapted from Ref. (27)]. The proapoptotic state is allowed when both DD tyrosines are dephosphorylated. The dominant proapoptotic state takes place when Y232 and/or Y291 is phosphorylated (some examples of possible dominant-negative scenarios are given). **(C)** A simplified illustration of Fas multi-signaling regulation by tyrosine phosphorylation switch. The non-death signaling triggered by activators such as soluble FasL (sFasL) is mediated by Src or Yes phosphorylation of the death domain tyrosines. The death signaling triggered by activators such as cross-linked FasL (cFasL) or membrane FasL (mFasL) is permitted by the dephosphorylation of the death domain tyrosines by SHP-1. **(D)** A diagram outlining pathologically relevant parameters leading to contexts that define the role of Fas signaling in human diseases (see text).

Unbound human Fas (hFas) DD comprises six α-helices (19) (Figure 1A). Fas DD can interact with many adaptors, including FADD (20–23). Resolved structures of Fas/FADD complex have identified some amino acids whose mutations could have pathological consequences (24–26). However, challenges remain in determining DD-mediated complex structure since the full-length receptor and the post-translational modifications, such as DD tyrosine phosphorylation, have yet to be taken into account.

## Death Domain Tyrosine Phosphorylation

While structural studies shed light into the DISC formation, an important question remained: “What gives the cue for Fas DD to form the DISC or a non-death-inducing signaling complex (NDISC)?” Because tyrosine Y232 and Y291 in hFas DD are phosphorylable (24), tyrosine phosphorylation is a prime candidate for the mechanism that determines when and which signaling complex is formed. Earlier studies reported that the Y291 of overexpressed hFas inhibited survival signals in mouse neutrophil (25). In the rat, whose Fas lacks the equivalent of Y291 of hFas (Figure 1A), Fas tyrosine phosphorylation associated with apoptosis in hepatocytes (26, 28). For nearly 20 years after the first report of hFas phosphotyrosine (pY), whether Y232 and/or Y291 phosphorylation was a switch for hFas multi-signaling had remained unclear, and the functions of each DD tyrosine had also remained unknown. The lag was due to the lack of practical pY mimetics for functional analyses and site-specific hFas pY detection.

Our recent study based on the analysis of evolution-guided Fas pY proxies and site-specific pY detection has revealed that DD tyrosine phosphorylation of hFas is the reversible antiapoptotic/pro-survival multi-signaling switch, namely, the DD tyrosine phosphorylation turns off the proapoptotic signal and turns on the pro-survival signals by dominantly inhibiting the DISC formation and apoptosis (Figure 1B) while promoting FasL-induced cell proliferation and migration (Figure 1C). We have also shown that pY232 and pY291 are regulated distinctly in some cancers (27).

## The Roles of Fas Death Domain Tyrosine Phosphorylation in Disease Etiology

That Fas DD pY dominantly inhibits Fas apoptotic signal and activates pro-survival signals invites a reassessment of our view about how Fas exerts its action in pathologies, such as cancer. A current opinion suggests that Fas signaling requires at least one wild-type *FAS* allele and that the signal transition from non-apoptotic to apoptotic signaling occurs when the Fas signal strength, exhibited by wild-type Fas protein, exceeds a threshold. This opinion is based on two reasons: (1) a mutated *FAS* allele that causes the loss of apoptotic function is often considered completely non-functional and (2) when Fas mutations are detected, tumors rarely have the loss of heterozygosity (18).

The threshold-based switch notion suggests that apoptotic signal requires two wild-type *FAS* alleles (strong signal) to reach its high threshold, while the threshold for the non-apoptotic signal is so low that it is attainable with one wild-type *FAS* allele (29). Based on the recent findings, the intermolecular and intramolecular “death-off” dominant inhibitory function of DD pY and its activating function for survival signals (27) suggest that the DD tyrosine phosphorylation is a highly efficient “on-off” multi-signaling switch. This information extends our views on Fas multi-signaling in diseases from threshold-based signaling switch to cover the concept that the apoptotic signal requires conditions that favor double dephosphorylation of the DD tyrosines, and the pro-survival signal is achievable in conditions that favor the phosphorylation of least one DD tyrosine.

## Regulators of Fas Death Domain Tyrosine Phosphorylation

### Src-Family Kinases

Src-family kinases (SFKs), including Src, Yes, Fyn, Blk, Yrk, Fgr, Hck, Lck, and Lyn, are protein tyrosine kinases that are preferentially expressed in different tissues (30, 31). Data from rodent models indirectly implied the role of Fyn and Yes as positive regulators of Fas-mediated apoptosis (32–36). Although, while some SFKs might play a proapoptotic role, they may not directly participate in Fas tyrosine phosphorylation. For example, the activation of human eosinophils led to a transient Fas tyrosine phosphorylation, followed by Lyn activation, which occurred concomitantly with Fas dephosphorylation (37). In fact, the phosphorylation of Fas by SFKs in cells had not been demonstrated till recently.

Studies of hFas in human colorectal cancer (CRC) cells have shown that Src and Yes play an important antiapoptotic and pro-survival roles in hFas signaling by phosphorylating hFas at Y232 and Y291 (27). The phosphorylation of Fas DD by Src and Yes leads to an inhibition of apoptosis and the enhanced cancer cell proliferation and migration, which are consistent with the oncogenic roles of these SFKs often reported in human cancers (38). The findings that (1) the levels of pY232 and pY291 increase in several types of cancer, including breast, ovarian, and colon cancers and (2) pY232 and pY291 levels appear to correlate with CRC progression (27) are in line with observations that the elevated Src and Yes levels correlate with advanced stages and metastatic potential of tumors and poor prognosis (39–42). In human glioblastoma multiforme (GBM), the Fas–Yes interaction and subsequent activation of PI3K/Akt pathway mediate glioblastoma invasion, and the Yes expression and phosphorylation of SFKs are present along with increased FasL expression in the tumor/host interaction zone in tumors of GBM patients (43). Additionally, Fas–Yes association leads to the activation of PI3K/Akt pathway and cell migration in human triple-negative breast cancer model (44). These observations support the role of SFKs in the Fas phosphorylation and tumor malignancy.

A point to keep in mind is the context under consideration. The roles of SFKs in Fas signaling and even the identity of the SFKs involved may differ appreciably in different tissues, disorders, or disease stages since expression profiles of kinases can vary significantly from one setting to another. For instance, while Src and Yes are key regulators of hFas phosphorylation in some solid tumors, this may not hold true for some hematopoietic malignancies where other oncogenic SFKs, such as Lck or Fgr are prominently present. Additionally, divergence in terms of regulatory specificity exists among model systems. For example, a non-conservative tyrosine phosphorylation site in Fas DD among primates and rodents (27) suggests diverse roles and identities of kinases that regulate Fas phosphorylation in different species. Therefore, extrapolating the regulation of Fas tyrosine phosphorylation switch from one species to another is likely to be inappropriate. Thus far, current information supports the notion that oncogenic SFKs, such as Src and Yes, are responsible for the tyrosine phosphorylation of DD of hFas and hence are positive regulators of Fas survival signals and negative regulators of Fas apoptotic signal.

### Src Homology Domain 2-Containing Tyrosine Phosphatase-1

Src homology domain 2 (SH2)-containing tyrosine phosphatase-1 (SHP-1), a protein tyrosine phosphatase, is predominantly present in hematopoietic cells and to a lesser extent in other cell types, including epithelial cells (45–48). Human SHP-1 appears to be a positive regulator of Fas-mediated apoptosis (27, 49) and a negative regulator of survival (25, 50), proliferation (51), and epithelial–mesenchymal transition (52). SHP-1 binding to hFas requires Y291 of Fas DD (25). In human CRC cells, it functionally opposes the effects of Src and Yes by dephosphorylating both pY232 and pY291, switching Fas from the antiapoptotic state to the proapoptotic/anti-proliferative state (Figure 1C) (27).

Notably, the rodent models demonstrate that the roles of SHP-1 depend on contexts, including tissues, diseases, and species. For example, Fas-mediated apoptosis was defective in the lymphoid organs but not in hepatocytes and thymocytes from SHP-1-deficient mice (49, 53). In mouse B cells, SHP-1 negatively regulates the DISC formation through its phosphatase activity on Vav1 (54). In rat granulocytes, SHP-1-CEACAM1 binding is important for downregulating FasL-induced apoptosis (53). The fact that rat Fas lacks the tyrosine shown to be the SHP-1-binding site in hFas (25) implies its distinct requirement for interacting with SHP-1 that may explain the different roles of rodent SHP-1 in Fas signaling. Like SFKs, identifying phosphatases and evaluating their roles in the regulation of Fas phosphorylation and signaling require a careful consideration of the contexts since the details of the signaling regulation can differ appreciably among species, tissues, disorders, or disease stages.

### Other Actors

Besides its cognate ligand and agonistic antibodies, other activators, including anticancer drugs and cytokines, can also activate Fas signaling (55–59). As we continue to unveil the control of pY-based mechanism of Fas multi-signaling switch, the roles of other molecules that may directly or indirectly influence the phosphorylation process of hFas DD, and the downstream Fas signaling will be clarified. For example, besides regulating the phosphorylation and non-death signaling of Fas (27), Yes also links Fas to EGFR and the PI3K/Akt pathways (44, 60), and thus PP2A (61). These actors, among others, are likely to participate in the mode of Fas signaling, at least indirectly. Further studies into the cross-talks between Fas and such actors, which are also drug targets (62–65), will not only further reinforce our understanding of context-dependent Fas signaling in human diseases but also aid in the design of efficient combinatorial therapies against diseases in which Fas is involved (66–68).

## Tyrosine Phosphorylation Switch System of Fas/FasL Signaling Pathways: A Bigger Picture

The involvement of tyrosine phosphorylation in Fas signaling has been well appreciated. In fact, the pY-based survival/apoptotic switch system also applies to other actors in Fas signaling network, with an important example being Caspase-8.

### Caspase-8

Human Caspase-8 (hCaspase-8) has at least three tyrosine phosphorylation sites. Phosphorylation of Y310, Y397, and/or Y465 (Y293, Y380, and/or Y488, respectively, in isoform B) suppresses Fas-mediated apoptosis (69–72). Additionally, prosurvival activators such as EGF can induce the phosphorylation of Caspase-8, which mediates its interaction with PI3K and cell adhesion and motility (73) and protects the cells from Fas-mediated apoptosis (69). The Caspase-8-mediated cell migration depends on pY380 but not the Caspase-8 activity or the death effector domain (74), thus separating non-apoptotic function of Caspase-8 from that of the receptor-mediated DISC formation path. Caspase-8 tyrosine phosphorylation may also participate in a positive feedback loop of caspase-8-induced Src activation and further promotes survival pathways (75). Additionally, pY310 of Caspase-8 promotes its interaction with SHP-1, which dephosphorylates Caspase-8, permitting its proapoptotic function (71).

Notably, Caspase-8 in rodents, including mouse, rat, guinea pig, and Chinese hamster lacks tyrosines at the positions equivalent to Y310 and Y380 in hCaspase-8. In mouse and guinea pig, it also lacks the tyrosine at the position equivalent to Y465 in hCaspase-8. Therefore, Caspase-8 multi-signaling regulation in rodents is likely to differ significantly from that of human, particularly regarding the dependence on tyrosine phosphorylation switch. Similar to Fas, this highlights the importance of careful consideration when applying the observed regulation and functions of Caspase-8 from one species to another. The lack of a tyrosine at the position equivalent to Y310 (SHP-1-binding site) of hCaspase-8 in rodent SHP-1 coincides with the observation that it participates in Fas signaling differently from human SHP-1. Thus, it is quite probable that, in different species, distinct sets of kinases and phosphatases regulate Fas multimode signaling system. This observation, along with other species-based variations in immune-related signaling (76, 77), serves as a reminder that, while the value of animal models is appreciable, particular attention should be given to the species-dependent details especially when designing targeted therapies. Notably, although the functional outcome among species may seem the same, the underlying mechanism might be very different.

That the SFK/SHP-1 phosphorylation-dephosphorylation mechanism applies to both Fas and Caspase-8, the main DISC components, based on information from human cells, suggests at least one common “survival-ON/death-OFF” switch system in the Fas signaling network and can help explain some observation of Fas-mediated outcome in human pathologies. For instance, hyperactivated Src and/or downregulation of SHP-1, such as in some cancers, may favor Fas DD and Caspase-8 tyrosine phosphorylation and thus the antiapoptotic/survival mode of Fas signaling.

## Outlooks

Interrupted apoptotic/non-apoptotic balance begets pathologies. Too little or too much of Fas-mediated apoptosis, as well as the rampant non-apoptotic activity of Fas can cause autoimmune diseases and cancer (3, 4, 12, 17, 43, 78, 79). Thus, several Fas-targeting therapeutics have been designed based on various strategies and are currently in different stages of development and clinical trials (Table 1). Understanding the apoptotic/non-apoptotic switch of Fas, which helps explain its pathological roles can help identify appropriate therapeutic choices. That said, how the apoptotic/non-apoptotic switch is set depends not only on modifications of the *FAS* gene but also on an intricate signaling network involving not only different forms of its ligand but also direct and indirect regulators, which varies according to genetic background. In the clinical aspect, parameters governing Fas multi-signaling may be divided into two main groups: Fas mutation-influenced parameters (MIPs) and regulator-influenced parameters (RIPs) (Figure 1D). Following examples illustrate the MIPs and RIPs in pathologies and their implications in context-dependent therapeutic design.

**Table 1:** **Examples of Fas/FasL-targeting drugs**.

Drug name	Design, target, and mode of action	Product stage	Companies
APO-010	APO-010 consists of three hFasL extracellular domains linked to a protein backbone comprising the dimer-forming collagen domain of human adiponectin. It targets cell surface Fas with an aim to induce Fas-mediated apoptosis of cancer cells (80)	– Phase I (completed): dose-finding study in patients with solid tumors (81) – Patient screening for Phase II is in progress for multiple myeloma (82, 83)	Oncology Venture ApS
ARG-098 (DE-098)	ARG-098 is a mouse/human chimeric monoclonal IgM antibody against hFas. It targets the Fas molecule, leading to apoptosis in synoviocytes (84)	Phase I/II for rheumatoid arthritis (85)	Janssen Biotech
			Santen
Novotarg	Novatarg is a bispecific antibody targeting CD20 and Fas. It is intended for inducing Fas-mediated apoptosis only in cells expressing CD20, which is an established target antigen for antibody-based immunotherapy in cancer (such as lymphoma) and B-cell-mediated autoimmune disease (86, 87)	Preclinical	Baliopharm
MOTI-1001	MOTI-1001 consists of the anticancer drug, paclitaxel, loaded in particles (Oncojans™) which are coated with Fas extracellular domain. It binds to FasL which often overexpressed on some cancerous cells. The binding can inhibit the invasion or proliferation, induced by FasL and trigger an active intracellular uptake process akin to phagocytosis. The ingested drug carrier forms a local drug reservoir inside the cancer cells and slowly releases paclitaxel, which binds to tubulin and interferes with the cell’s cytoskeleton (88, 89)	Preclinical study for ovarian cancer (89)	Biomoti
APG 101 (Apocept)	APG101 consists of the extracellular domain of hFas linked to the Fc domain of human IgG1 (Fas-Fc). It binds FasL and therefore inhibits the activation of Fas signaling (90, 91)	– Phase II (completed) for glioblastoma (90) – Phase I (completed) for myelodysplastic syndromes (MDS)	Apogenix

### Autoimmune Lymphoproliferative Syndrome

Autoimmune lymphoproliferative syndrome (ALPS) is often affected by MIPs since Fas mutations, mostly in DD, are the common cause of the disease (17). Some Fas mutations cause defects in the binding to FADD, inhibiting the DISC formation and apoptosis. Other mutations may promote the NDISC formation, which may depend on the regulation of DD tyrosine phosphorylation. As such, ALPS cases that fall into different contexts of MIPs can respond differently to the same intervention. Thus, therapeutic approaches tailored for ALPS in context-dependent manners are desirable.

### Systemic Lupus Erythematosus

Systemic lupus erythematosus (SLE) illustrates multi-faceted MIPs and RIPs that involve both the downregulation of apoptosis and the upregulation of migration mediated by Fas. MIPs in SLE include *FAS* gene polymorphisms and mRNA editing. Fas gene polymorphisms have been associated with susceptibility to SLE (92), although, the consequence of the polymorphisms on the protein’s function is unclear. Recently, a *FAS* mRNA mutation caused by an mRNA editing has been reported in SLE patients (93). While the *FAS* gene mutation is absent in the genomic DNA, the mRNA editing leads to the production of an apoptosis-defective truncated Fas protein with a frameshift at the end of DD especially in T cells of the SLE patients (93). Meanwhile, RIPs in SLE include the involvement of Lyn and the expression levels of soluble Fas (sFas) and sFasL. The loss of function of Lyn, which is implicated in the development of SLE (94), can also dampen Fas-mediated apoptosis (37). Elevated levels of sFas and sFasL of a significant number of SLE patients (60, 95) are also associated with the disease flare (96). The sFasL is implicated in the trafficking of T helper 17 lymphocytes to inflamed organs (12). Meanwhile, it is unclear whether the elevated level of sFas in SLE patient prevents Fas-mediated apoptosis, as previously suggested (95) or counteracts the effect of elevated sFasL level in SLE patients. How these RIPs influence the Fas signaling switch is yet to be clarified. And as such, different SLE cases influenced by the different context of MIPS and RIPs will have to be handled accordingly.

### Glioblastoma Multiforme

The progression of GBM, where Fas mutations are uncommon (97), involves RIPs that promote non-apoptotic signaling of Fas, such as SFK and FasL expression. An elevated SFK activity in GBM (98) can promote NDISC and GBM cell invasion (43). FasL also contributes toward the invasive phenotype of glioblastoma cells (99). In GBM cases, where an SFK that promotes Fas DD phosphorylation (e.g., Src or Yes) is hyperactivated, the intervention should thus be aimed at inhibiting NDISC that can promote cancer progression. Such interventions may be achieved by preventing FasL from binding to Fas and thus preventing the Fas activation and/or by specifically inhibit the activities of Src and Yes and thus preventing Fas DD phosphorylation. In agreement with this view, a drug that prevents FasL from activating Fas non-apoptotic signals shows promise in reducing GBM progression (90). One may expect that integrating this approach with inhibiting Src and Yes (100), can further improve therapeutic success for GBM.

### Myelodysplastic Syndrome

A hyper-apoptotic signaling is significant in myelodysplastic syndromes (MDS) (101), where RIPs, such as overexpression of Fas (79) and SHP-1 (102) are prominent. When SHP-1 (the suppressor of Fas DD phosphorylation) is upregulated, inhibiting the rampant apoptosis, such as by blocking FasL or SHP-1, should be beneficial. In agreement with this view, preventing FasL from activating Fas apoptotic signal in MDS seems promising (91). It can also be envisaged that combining this approach with SHP-1 inhibition may further improve therapeutic success for MDS.

Overall, the findings discussed here emphasize the importance of examining Fas multi-signaling while considering different contexts that can influence Fas signaling switch. A context-oriented understanding of Fas multi-signaling will allow the efficient design of Fas-related therapeutic strategies.

## Author Contributions

All authors listed have made substantial, direct, and intellectual contribution to the work and approved it for publication.

## Conflict of Interest Statement

The authors declare that the research was conducted in the absence of any commercial or financial relationships that could be construed as a potential conflict of interest.
